# Cor triatriatum dexter as an incidental finding due to symptomatic bicuspid aortic valve stenosis

**DOI:** 10.1177/02676591231182584

**Published:** 2023-06-06

**Authors:** Evangelos Anastasakis, Vasilios Grosomianidis, Paschalis Tossios, Adnan Charaf, Mazin A.I. Sarsam, Georgios T. Karapanagiotidis

**Affiliations:** 1 4915 St George’s University of London, London, UK; 2 1333 Ashford and St Peter’s NHS Foundation Trust, Chertsey, UK; 3 37788 AHEPAUniversity Hospital, Thessaloniki, Greece; 4 Department of Cardiothoracic Surgery, 156611 St George’s Hospital, London, UK

**Keywords:** Cor triatriatum dexter, cor triatriatum, congenital heart disease, aortic valve disease, bicuspid aortic valve

## Abstract

Cor triatriatum is a rare congenital heart defect in which a thin, fibro-muscular membrane divides the left or right atrium into two chambers resulting in a triatrial heart. Subdivision of the left atrium named cor triatriatum sinister (CTS), is the more common form, whereas the right atrial equivalent called cor triatriatum dexter (CTD) is rarer. They account for up to 0.4% and 0.025% of the burden of congenital heart disease respectively. We present the case of CTD found incidentally with transthoracic echocardiography for a patient who underwent aortic valve replacement for symptomatic bicuspid aortic valve stenosis.

## Introduction

Cor triatriatum dexter (CTD) is an extremely rare congenital heart defect, in which the right atrium is divided into two chambers by a thin, fibro-muscular membrane. There is a resulting triatrial heart. It is rarer than its left atrial counterpart cor triatriatum sinister (CTS) and has an estimated incidence of around 0.025% of all congenital heart disease.^1^

CTD results from a persistence of the embryonic right venous valve. In embryo, the right and left venous valves, guard the sinoatrial orifice, through which blood flows from the sinus venosus to the primitive atrium.^2^ In normal development, the left venous valve forms part of the septum secundum and the right venous valve is reabsorbed between the 9^th^ and 15^th^ week of gestation, leaving behind the Eustachian valve, Thebesian valve and terminal crest.^2,3^ Incomplete resorption of the right venous valve can occur to various degrees: mild forms can lead to prominent Eustachian and Thebesian valves; a prominent terminal crest; or a Chiari network.^2^ Complete persistence of the right venous valve will sub-divide the right atrium causing CTD.^3^

Clinical presentation in CTD is dictated by how this fibro-muscular membrane interferes with venous return. Should it be obstructive, patients present with signs of right heart failure, whereas non-obstructive lesions are usually asymptomatic with CTD being found incidentally on imaging.^4^ CTD is often associated with other right-sided congenital heart defects including pulmonary artery stenosis and atresia, atrial septal defects, tricuspid valve defects and Ebstein’s anomaly.^5^

Diagnosis is via transthoracic or transoesophageal echocardiography; however magnetic resonance imaging can also be used.^5^ Asymptomatic patients do not require intervention and can be monitored, whereas symptomatic patients should undergo surgical resection of the fibro-muscular membrane or percutaneous transluminal correction.^4,6^

## Case report

We present the case of a 56 year-old male who presented with exertional dyspnoea and chest pain. Examination revealed a grade IV aortic, ejection systolic murmur. Past medical history was significant for diabetes with no congenital cardiac history. Transthoracic echocardiography revealed severe aortic stenosis secondary to a bicuspid aortic valve. The patient underwent an elective aortic valve replacement (AVR). Median sternotomy was performed and AVR was carried out using cardiopulmonary bypass and cardioplegic cardiac arrest. A bicuspid aortic valve with fusion of the right and left coronary leaflets was visualised. This was replaced using a 23 mm, Edward’s Inspiris RESILIA, tissue prosthetic valve, due to the patient wishing to avoid life-long warfarin. There were no complications to note.

**Figure 1.**
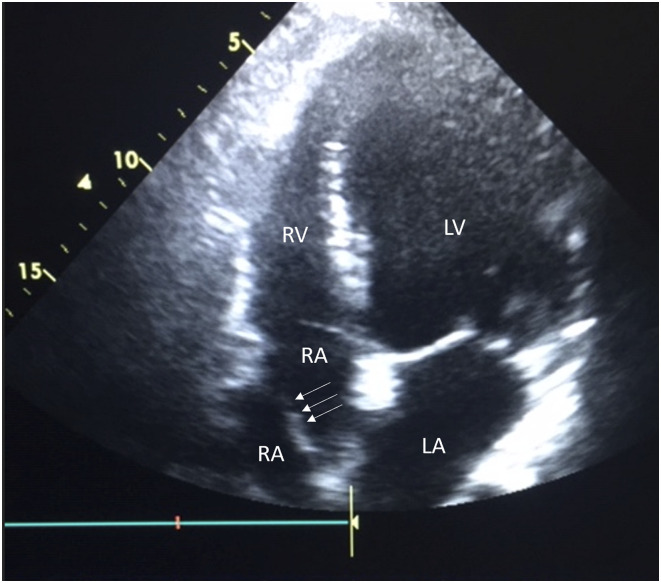
Transthoracic echocardiogram showing CTD. A membranous defect extending across the diameter of the right atrium and attaching to the atrial septum is visible.

Post-operative results were good with resolution of symptoms. Transthoracic echocardiography on day five post-operatively, showed significant abolition of the mean gradient. Also visible, was a transverse membrane, attached to the atrial septum, separating the right atrium into two distinct compartments (Figure 1). This was diagnostic for CTD. As the patient was asymptomatic with normal myocardial function and central venous pressure, no further investigation or management was required. He remained asymptomatic at 12 month follow-up.

## Discussion

Due to its often-asymptomatic nature, CTD may first appear at pre-operative assessment for a separate cardiac pathology. This is significant, as the fibro-muscular membrane is a potential source of obstruction for catheter-based cardiac procedures.^7^ Moreover, CTD may represent a challenge when instituting cardiopulmonary bypass during cardiac surgery, as the right atrium may prove difficult to cannulate. Therefore, if CTD has not been identified pre-operatively, peri-procedural issues may arise without an obvious cause. Notably, in our case, we were successful in cannulating the right atrium without any issues.

Having identified CTD, questions arise regarding management. Asymptomatic patients are usually monitored with no intervention, and this was the approach we took. However, it is necessary to recognise that cases of CTS and CTD can present as cardioembolic stroke and pulmonary embolism (PE) respectively.^8–10^ The mechanism being that the membrane may act a site of thrombus formation.^11^ Conversely, it may in fact be protective against PE and stroke by acting as an anatomical filter and barrier.^12,13^ When considering the indication for long-term anticoagulation, our opinion is that in the absence of cardiac arrhythmias or past thromboembolic events, patients should be monitored. It is noteworthy, that arrhythmias are a known complication of cardiac surgery and, we had a low threshold to anti-coagulate, should an arrhythmia have developed.^14^

For symptomatic CTD, currently surgical resection is recommended with the alternative being percutaneous transcatheter disruption.^2,4,7^ The latter is less preferable as the ability to effectively visualise the anatomy is limited and this is important when there are other associated anatomical defects that require concomitant correction.^7^ These are most frequently atrial septal defects.^7^

## Conclusion

CTD is an extremely rare congenital heart defect which often presents asymptomatically. In these cases, it is important to consider that the potentially fibro-muscular membrane may obstruct percutaneous catheter-based procedures or cannulation of the right atrium during institution of cardiopulmonary bypass.
